# Bridging substrate intake kinetics and bacterial growth phenotypes with flux balance analysis incorporating proteome allocation

**DOI:** 10.1038/s41598-020-61174-0

**Published:** 2020-03-09

**Authors:** Hong Zeng, Aidong Yang

**Affiliations:** Department of Engineering Science, University of Oxford, Parks Road, Oxford, OX1 3PJ UK

**Keywords:** Biochemical networks, Computer modelling, Systems analysis

## Abstract

Empirical kinetic models such as the Monod equation have been widely applied to relate the cell growth with substrate availability. The Monod equation shares a similar form with the mechanistically-based Michaelis-Menten kinetics for enzymatic processes, which has provoked long-standing and un-concluded conjectures on their relationship. In this work, we integrated proteome allocation principles into a Flux Balance Analysis (FBA) model of *Escherichia coli*, which quantitatively revealed potential mechanisms that underpin the phenomenological Monod parameters: the maximum specific growth rate could be dictated by the abundance of growth-controlling proteome and growth-pertinent proteome cost; more importantly, the Monod constant (*K*_*s*_) was shown to relate to the Michaelis constant for substrate transport (*K*_*m*,*g*_), with the link being dependent on the cell’s metabolic strategy. Besides, the proposed model was able to predict glucose uptake rate at given external glucose concentration through the size of available proteome resource for substrate transport and its enzymatic cost, while growth rate and acetate overflow were accurately simulated for two *E. coli* strains. Bridging the enzymatic kinetics of substrate intake and overall growth phenotypes, this work offers a mechanistic interpretation to the empirical Monod law, and demonstrates the potential of coupling local and global cellular constrains in predictive modelling.

## Introduction

Understanding the growth of microbial cultures occupies a central place in the study of microorganisms. How growth rate varies with internal traits or external conditions greatly affects the choice of process-level parameters such as temperature, medium composition in cell cultures for a specific purpose and the strategy for the modulation of delicate cellular attributes such as enzyme activity, pathway diversity and regulatory systems.

Ever since Jacques Monod correlated the specific growth rate (*λ*) with extracellular substrate concentration ([*g*]) via two kinetic parameters, the maximum specific growth rate *λ*_*max*_ and the Monod constant *K*_*s*_, the Monod equation $\lambda ={\lambda }_{max}\frac{[g]}{[g]+{K}_{s}}$^1^ becomes the best-known microbial growth kinetics for describing various bioprocesses. Other growth models (reviewed in^2^) are less prevalent due to more sophisticated formulation and insufficient experimental validation. Despite its unstructured and empirical nature^3^, the Monod equation generally renders satisfactory results in terms of fitting substrate consumption and growth rate profile for a wide range of microorganisms and culture conditions. More recent studies have shown that growth kinetic constants (in Monod terms, *λ*_*max*_ and *K*_*s*_) are actually a function of the culture history (e.g. sludge ages) and community composition^4,5^. In fact, twenty-years ago Kovarova-Kovar and Egli explicitly pointed out that cells could change their growth kinetics via adaptation, therefore a single set of kinetic parameters is not able to represent such variable properties^2^. The notion of variable growth kinetics is further supported by experimental evidence showing the evolution of *K*_*s*_^6,7^, ‘intrinsic’ and ‘extant’ *λ*_*max*_^8^ and the ${\lambda }_{max}-{K}_{s}$ relationship^9^.

In addition to the Monod-type empirical models, the development of metabolic network reconstructions and the associated analytical approach, namely Flux Balance Analysis (FBA) facilitates the study of growth phenotypes at the metabolic flux level^10^. A FBA model is established based on the known metabolic reaction stoichiometry and gene-enzyme relationship. It is inherently mechanistic and provides more insight of cellular physiological properties compared to an empirical growth model. More importantly, the general objective of FBA is to predict the maximum growth rate at given culture conditions and possibly genetic modifications^11^. If the prediction of growth rate and intracellular metabolic fluxes were sufficiently accurate, FBA would become a very useful tool for engineering efficient microbial systems. Essentially, the accuracy of the flux prediction in FBA is governed by constraints. One typical example is setting uptake limits for oxygen and glucose exchange fluxes to predict aerobic acetate formation (namely the overflow metabolism) in *Escherichia coli*^12^, where acetate was predicted to excrete when the system was limited by oxygen uptake rate. However, it has been proposed and validated that the aerobic acetogensis in *E. coli* is the result of an efficient proteome allocation strategy for rapid growth^13,14^. This example suggests that setting hard limitations on uptake fluxes could be a rather arbitrary and problematic treatment when there is a lack of theoretical basis, not to mention that the boundaries of myriad intracellular fluxes are often difficult to determine reliably. In principle, using experimentally determined flux values as lower and/or upper bounds could help FBA to compute more realistic flux distributions. This leads a dilemma between the overall accuracy of the simulation and the predictive power of the model (e.g. fluxes with hard-set boundaries are in a sense “weakly” predicted). Progress in incorporating macromolecular expressions^15^ and intracellular resource allocation^16–19^ with the classic FBA effectively alleviates the need for setting subtle uptake boundaries and improves the accuracy and scope of the predicted growth phenotypes. For instance, Resource Balance Analysis (RBA)^18^ eliminates the need of defining uptake bounds of substrates by relating the enzyme efficiency with the concentration of extracellular nutrients. Multi-scale models of Metabolism and macromolecular Expression (ME-models)^20–22^ enable the prediction of substrate uptake rates by incorporating growth rate-dependent demand functions and limited macromolecular synthesis machineries, e.g. limited ribosomal translation rate, limited mRNA catalytic rate and limited RNA polymerase transcription rate. Despite these successes, the prediction of substrate uptake rate in general highly relies on the accuracy of the enzyme kinetic parameters adopted in the model, most of which are not readily available at the genome scale^23^.

In this work, we explore the potential links between the empirical Monod growth kinetics and the FBA-based metabolic modelling, with the latter to reveal the underlying biological mechanisms governing the phenomenological parameters of the former. Using two *E. coli* strains (NCM3722 and ML308) as case studies, we investigate how the integration of the proteome allocation principles into FBA would help depict the intriguing multi-scale mechanisms that govern the various growth phenotypes under a wide range of growth conditions. We firstly divided the overall proteome into coarse-grained functional sectors, i.e. carbon scavenging (C), energy generation (E), biomass synthesis (BM) and growth-independent offset (Q) sectors^19,24^. The size of each sector is dictated by the magnitude of the metabolic flux the sector processes and the corresponding proteome cost per unit flux. All individual proteome sectors were assembled together into a global proteome allocation constraint^24–26^. Furthermore, we parameterized a correlation between the enzyme cost for carbon transport and the abundance of carbon source. Applying these modelling concepts and parameterisation in FBA simulations, we were able to predict the glucose uptake rate, growth rate and the acetate overflow upon varying extracellular glucose level. Our theoretical model also predicted a step change in *K*_*s*_ after the onset of the acetate excretion. Further analysis of the model suggests that this variation originates from the change in cell’s metabolic strategy, which coincides with the previous notion that cells possess variable growth kinetics due to adaptation to the environment^2^. Besides, *λ*_*max*_ is shown to be controlled by the abundance of growth-controlling proteome and the proteome cost per unit increase of growth rate. Finally, we propose that a proper connection of local and global physiological constraints may be essential for improving the predictive power of FBA models.

## Results

### Proteomic fraction occupied by the carbon-scavenging sector

**Figure 1:**
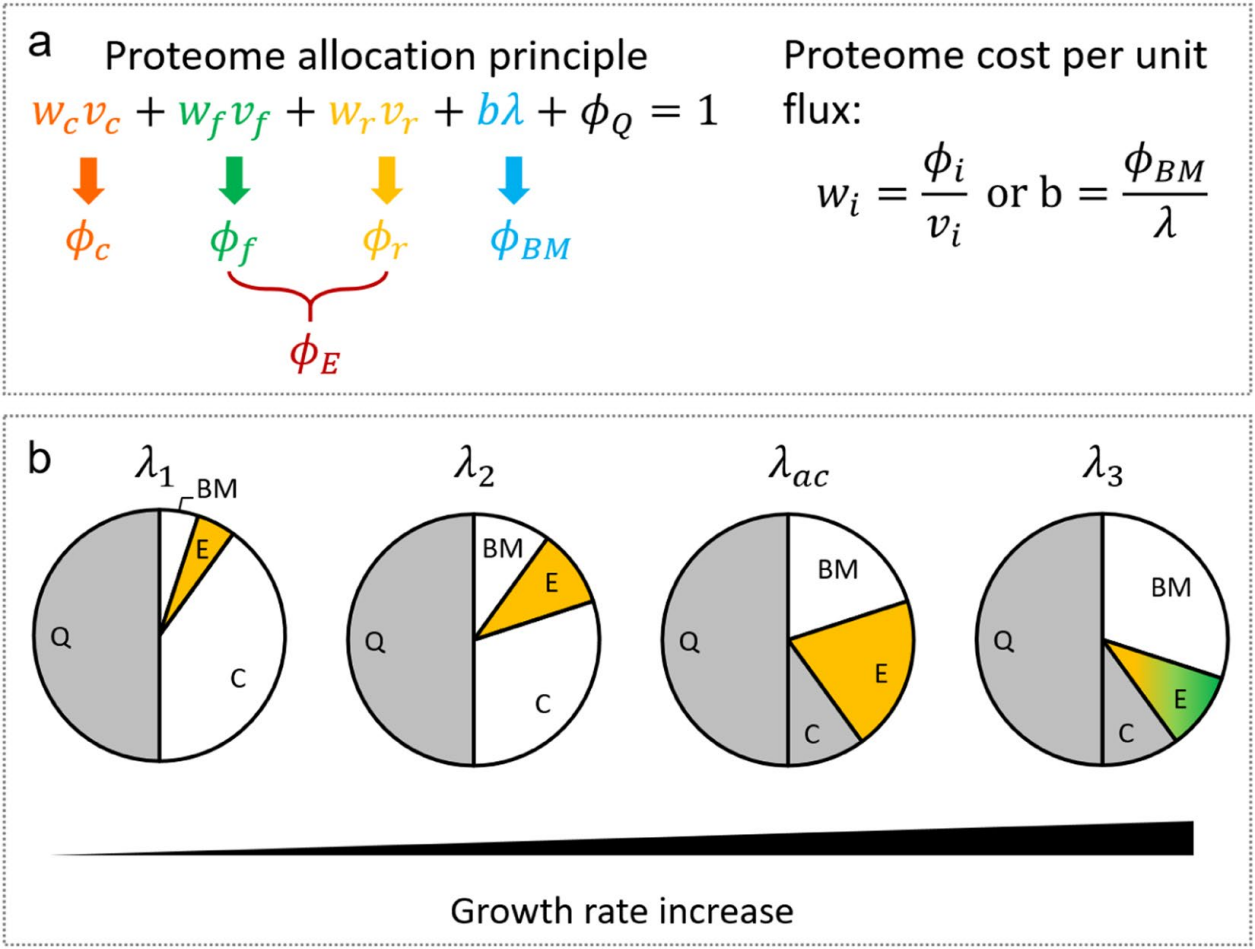
Schematic diagram of the proteome allocation model. **(a)** The overall proteome comprises four sectors, including carbon-scavenging ${\phi }_{C}$, energy biogenesis ${\phi }_{E}$, biomass synthesis ${\phi }_{BM}$ and growth-independent offset ${\phi }_{Q}$. The enzymatic cost per unit flux of each sector is calculated by the ratio between proteome abundance and the corresponding flux. **(b)** The evolution of proteome component at increased growth rate. Before acetate overflow, BM and E sectors increase with growth rate while the C sector continuously declines; The E sector comprises only respiration (marked in yellow). At $\lambda ={\lambda }_{ac}$, ${\phi }_{E}={\phi }_{r}$ reaches the highest value. At higher growth rates, the E sector starts to decline to save space for the increase of the BM sector and becomes a mixture of fermentation and respiration (marked with a mixture of yellow and green); the C sector reaches its lower bound and stays constant.

Following the treatment in several recent studies on proteome allocation^14,19,25–27^, we divide the overall cell proteome into four coarse-grained functional sectors: carbon-scavenging sector C that comprises enzymes for importing extracellular carbohydrate, energy biogenesis sector E that consists of enzymes used for respiration and (aerobic) fermentation, biomass synthesis sector BM that accounts for ribosomal proteins and enzymes carrying anabolic fluxes, and an offset sector Q that contains other proteins whose abundance do not vary with the fluxes in the above three sectors (Fig. 1a). The summation of the fractions of all sectors (i.e. ${\phi }_{C},{\phi }_{E},{\phi }_{BM},{\phi }_{Q}$) equals to one

1 $${\phi }_{C}+{\phi }_{E}+{\phi }_{BM}+{\phi }_{Q}=1$$

Let the maximum amount of proteome attainable to C, E and BM sectors be ${\phi }_{max}^{g}$, which corresponds to the minimum size of the Q sector, ${\phi }_{Qmin}$. For cells striving for maximum growth,

2 $${\phi }_{C}+{\phi }_{E}+{\phi }_{BM}=1-{\phi }_{Qmin}={\phi }_{max}^{g}$$

This global constraint is coupled to another proteome constraint that was established previously as a key mechanism governing the overflow metabolism in *E. coli*^14,28^

3 $${\phi }_{E}+{\phi }_{BM}\le {\phi }_{max}^{o}$$

**Figure 2:**
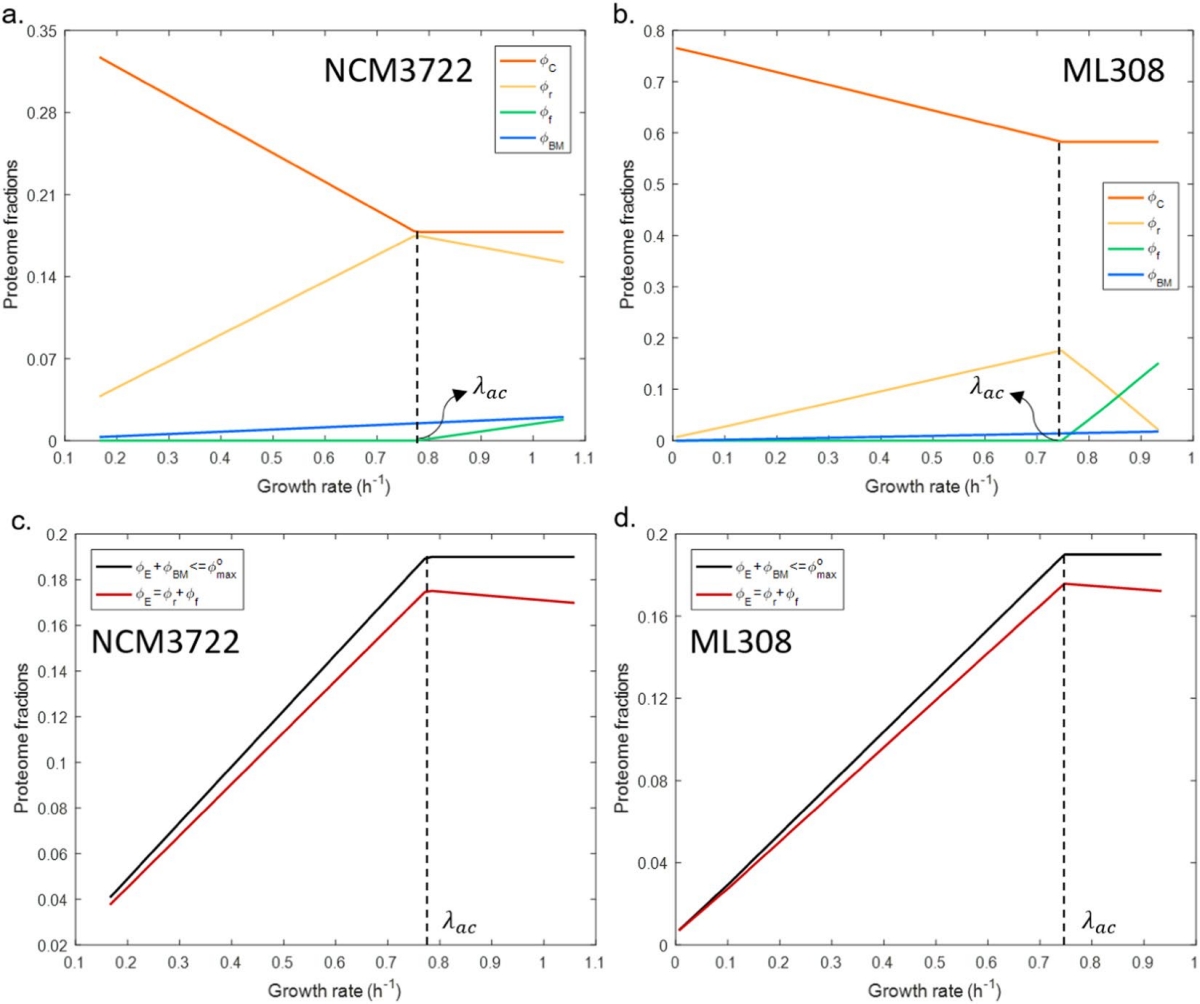
Simulated distribution of proteome sectors ${\phi }_{C}$, ${\phi }_{r}$, ${\phi }_{f}$ and ${\phi }_{BM}$ against different growth rates for *E. coli* NCM3722 **(a)** and ML308 **(b)**; and simulated change of the E sector $({\phi }_{E})$ and summation of E and BM sectors $({\phi }_{E}+{\phi }_{BM})$ for NCM3722 **(c)** and ML308 **(d)**. ${\phi }_{r}$ and ${\phi }_{f}$ are E sector components. The dashed line indicates the onset of acetate overflow. *λ*_*ac*_ is the threshold growth rate, above which acetate overflow occurs.

${\phi }_{max}^{o}$ denotes the maximum amount of proteome attainable to energy production and biomass synthesis. Equations (2 and 3) were used to derive two concise proteome allocation constraints (Eqs. (16 and 17), Methods), which were integrated into the *E. coli* metabolic network as additional rows. The resulted proteome allocation-constrained FBA model forms a mixed integer liner programing (MILP) problem (Supplementary Text). Determination of model parameters is detailed in Supplementary Text. FBA simulations were performed using the integrated model with extracellular glucose concentration as input and maximising cell growth as the objective function. Simulated optimal growth rate (*μ)*, carbon uptake rate (*v*_*c*_), acetate production rate (*v*_*f*_) and tricarboxylic acid cycle flux (*v*_*r*_) at *μ*, along with proteome cost parameters (defined in Eqs. (14–16)) were used to calculate the distribution of proteome sectors (defined in Eq. (2)) at different growth rates (Fig. 2a,b). Simulation results show that ${\phi }_{C}$ gradually decreases as growth rate increases until it reaches a minimum level $({\phi }_{Cmin})$, which is equal to the gap between ${\phi }_{max}^{g}$ and ${\phi }_{max}^{o}$:

4a $${\phi }_{C} > {\phi }_{Cmin},\,when\,\lambda  < {\lambda }_{ac}$$

4b $${\phi }_{C}={\phi }_{Cmin}={\phi }_{max}^{g}-{\phi }_{max}^{o},\,when\,\lambda \ge {\lambda }_{ac}$$

The growth rate at which ${\phi }_{C}$ reaches its lower bound is corresponding to the threshold growth rate *λ*_*ac*_, above which acetate overflow occurs (Fig. 2a,b). When growth rate exceeds *λ*_*ac*_ the composition of ${\phi }_{E}$ changes from the enzymes for pure respiration to those for a combination of fermentation and respiration (Fig. 1a). The predicted decline of ${\phi }_{C}$ is supported by the observed upregulation of catabolic genes under carbon limitation^25^. We did not find experimental evidence for a non-zero ${\phi }_{Cmin}$. However combining Eqs. (2 and 3) gives ${\phi }_{C}\ge {\phi }_{max}^{g}-{\phi }_{max}^{o}$, which in theory implies a positive value of ${\phi }_{Cmin}$ given ${\phi }_{max}^{g} > {\phi }_{max}^{o}$.

### Predictions of glucose uptake rate and the acetate overflow

It has been shown that the incorporation of Eq. (3) into a metabolic model of *E. coli* enables the prediction of the acetate overflow at $\lambda \ge {\lambda }_{ac}$^28^. Assuming a linear relationship between a proteome fraction and the metabolic flux it processes^25^, the C sector could be modelled as

5 $${\phi }_{C}={w}_{c}{v}_{c}$$

where *v*_*c*_ is the carbon uptake flux. The linear coefficient *w*_*c*_ denotes the enzyme cost per unit carbon influx, which has been proposed to be a function of the environmental substrate levels, derived from the Michaelis-Menten equation^19^

6 $${w}_{c}={w}_{c,0}(1+\frac{{K}_{m,g}}{[g]})$$

**Figure 3:**
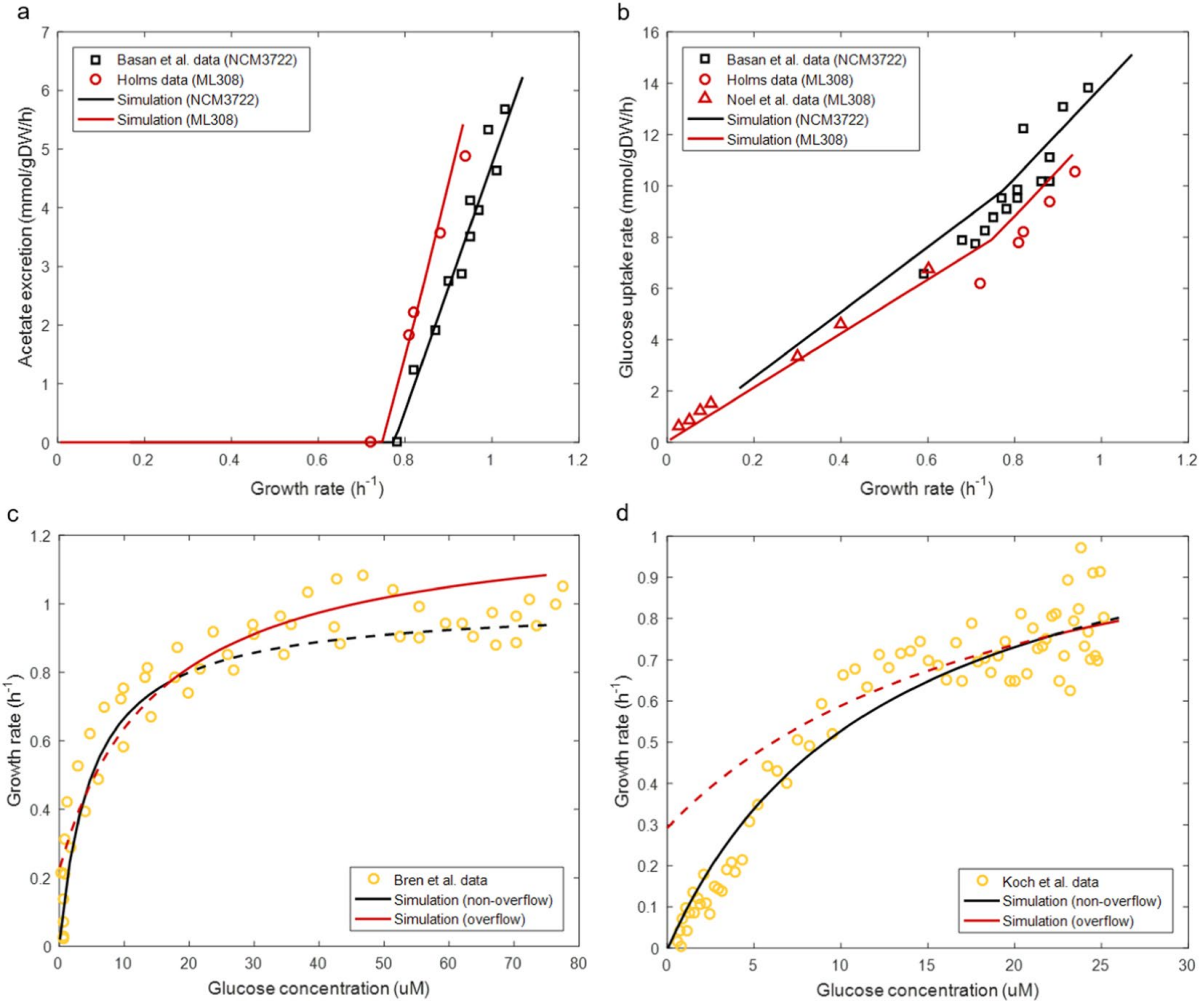
Comparison between the model simulation and experimental data of the growth of *E. coli* in glucose-limited cultures. **(a)** Comparison between predicted and measured acetate excretion rate. Experimental data for NCM3722 were obtained from Fig. 1 of ref. ^14^; experimental data for ML308 were obtained from Table 7 of ref. ^52^. **(b)** Comparison between predicted and measured glucose uptake rate. Experimental data for NCM3722 were obtained from Fig. 3B of ref. ^19^; experimental data for ML308 were obtained from Table 7 of ref. ^52^ and Fig. 3b of ref. ^53^. **(c)** Comparison between predicted and measured specific growth rate against glucose concentration profile for NCM3722. Experimental data were obtained from SI Fig. 1 of ref. ^51^. **(d)** Comparison between predicted and measured specific growth rate against glucose concentration profile for ML308. Experimental data were obtained from Fig. 1 of ref. ^34^. Solid lines in **(c**,**d)** are actual model predictions; dashed lines are presented to show the model prediction in the region where the model is not applicable. Simulation results of optimal growth rate, glucose uptake rate and acetate production rate were obtained in FBA simulations of the proteome allocation-integrated metabolic model. In all simulations, the extracellular glucose concentration was specified as input and the growth rate was maximised.

The constant *w*_*c*,0_ represents the minimum enzyme cost per unit carbon influx; [*g*] is the glucose concentration, where glucose is taken as a representative substrate; *K*_*m,g*_ is the Michaelis constant. We determined the value of *w*_*c*,0_ using strain-specific cell culture data (Supplementary Text); with reported values of *K*_*m,g*_ we were subsequently able to compute *w*_*c*_ as a function of [*g*] (Supplementary Eqs. (S36 and S37)). Coupling Eq. (6) with the proteome allocation constraints (Eqs. (16 and 17)), the glucose uptake rate, acetate production rate and optimal growth rate of two *E. coli* strains were predicted by specifying [*g*] (as opposed to setting glucose influx as pre-determined input^12^) and maximizing the growth rate. Simulation results agree well with the experimental data (Fig. 3a–d).

### *λ*_*max*_ is a function of proteome resource and proteome cost

Combining the proteome allocation constraints and the established ${w}_{c}-[g]$ correlation (Eq. (6)) with additional linear assumptions for the ${v}_{c}-\lambda$ pair (Supplementary Text), we have derived

7 $${\lambda }_{max}={\phi }_{growth}/{p}_{growth}$$

${\phi }_{growth}$ represents the maximal fraction of the growth-controlling proteome, and ${p}_{growth}$ is the proteome cost per unit increase of growth rate. Equation (7) is in line with the previous proposal that *λ*_*max*_ is closely related to the protein synthesis mechanism^4^. During non-overflow growth $(\lambda  < {\lambda }_{ac})$, we show that (Supplementary Eqs. (S16–S21))

8 $${\phi }_{growth}={\phi }_{max}^{g}-\sum _{i}{\phi }_{i,atpm}$$

9 $${p}_{growth}=\sum _{i}{p}_{i}$$

${\phi }_{i,atpm}$ is the proteome fraction of sector *i* occupied by non-growth-associated maintenance; *p*_*i*_ is the proteome cost of sector *i* per unit increase of growth rate; *i* represents C, E, or BM sector. During overflow growth $(\lambda \ge {\lambda }_{ac})$, we show that (Supplementary Eqs. (S22–S24))

10 $${\phi }_{growth}={\phi {\prime} }_{c}$$

11 $${p}_{growth}={p}_{c}$$

${\phi {\prime} }_{c}$ denotes the fraction of the proteome allocable to the carbon-scavenging sector (adjusted with an off-set to ${\phi }_{Cmin}$, see Supplementary Eq. (S22)). Equations (10 and 11) imply that in the overflow region ${\lambda }_{max}$ can be dictated solely by the characteristics of the C sector.

The above results suggest two ways to improve cell growth: increase ${\phi }_{growth}$ or decrease *p*_*i*_. ${\phi }_{growth}$ could be elevated by reducing maintenance or eliminating the expression of useless proteins. However, it might be difficult to directly modify ${\phi }_{growth}$ as it is more or less an intrinsic feature of a cell. Reducing *p*_*i*_ could be more realistic in synthetic biology. In this work *p*_*c*_ could gradually reduce upon the decline of *w*_*c*_ at increased growth rate (Supplementary Eqs. (S19 and S23) and Supplementary Fig. S1). *p*_*E*_ was shown to change from positive to negative due to varied ${\phi }_{E}$ against growth rate correlation before and after *λ*_*ac*_ (Fig. 2c,d). The variability of *p*_*i*_ could result from two mechanisms: the cell upregulates catabolic genes under carbon limitation (leads to variable *p*_*c*_) and changes its metabolic strategies under different culture conditions (leads to variable *p*_*E*_). The latter is governed by the presence of alternative pathways. We could infer that if a cell carries multiple pathways of a similar biological function (e.g. energy biogenesis) but with distinct enzyme (or protein) costs, the cell might be able to improve its growth via directing more metabolic flux to the enzymatically low-cost pathways. This could help design synthetic microorganisms with potentially higher robustness and fitness to the growth environment. A recent study also predicted that improved enzyme activity of energy pathways leads to a higher growth rate^29^, which supports the above suggestions.

### *K*_*s*_ depends on the interplay between fractions of proteome sectors and affinity of transport enzymes

While *λ*_*max*_ unambiguously refers to the highest specific growth rate a cell can achieve, the biological meaning of *K*_*s*_ is less clear. Often taken as a mathematical analogy to the Michaelis-Menten enzyme kinetics, the Monod constant *K*_*s*_ seems to share some link with the Michaelis constant for substrate transport *K*_*m,g*_. This might explain why the Monod growth kinetics is sometimes mistermed as “Michaelis-Menten kinetics” for describing growth-associated bioprocesses^2^. While the derivation of the Monod equation is strictly empirical^30^, Monod himself raised interesting comments between *K*_*s*_ and *K*_*m,g*_: (i) ‘the value of *K*_*s*_ should be expected to bear some more or less distant relation to the apparent dissociation constant of the enzyme involved in the first step of breakdown of a given compound’; (ii) ‘since a change of conditions affecting primarily the velocity of only one rate-determining step will, in general (but not necessarily), be only partially reflected in the overall rate, one might expect *K*_*s*_ values to be lower than the corresponding values of the Michaelis constant of the enzyme catalyzing the reaction’^1^. Essentially Monod suggested that *K*_*s*_ could be a function of *K*_*m,g*_ and that *K*_*s*_ should be generally smaller than *K*_*m,g*_. In the past twenty years several attempts have been made to explore the physical meaning of *K*_*s*_. One interpretation is that 1/*K*_*s*_ relects the overall affinity of a cell to a substrate^2^. A later study suggests that *K*_*s*_ is a function of the overall change of free energy of the microbial growth process^31^. More recently, *K*_*s*_ was related to *K*_*m,g*_ via investigating the control of the transport step on the specific growth rate^30^. In this work, we explicitly derived that *K*_*s*_ relates to *K*_*m,g*_ via a proportional factor *δ* (Supplementary Eqs. (S15) and (S26–S29))

12 $${K}_{s}=\delta {K}_{m,g}$$

13 $$\delta =\{\begin{array}{ll}\frac{{p}_{c}}{{\sum }_{i}{p}_{i}} < 1, & when\,\lambda  < {\lambda }_{ac}\\ 1, & when\,\lambda \ge {\lambda }_{ac}\end{array}$$

The discrete feature of *δ* leads to a (upward) step change of *K*_*s*_ at the acetate switch (${K}_{s,NCM3722}=5\,\mu M$ and ${K}_{s,ML308}=12\,\mu M$ for $\lambda  < {\lambda }_{ac}$; ${K}_{s,NCM3722}=15\,\mu M$ and ${K}_{s,ML308}=20\,\mu M$ for $\lambda \ge {\lambda }_{ac}$), which perfectly validates Monod’s proposition that ${K}_{s}=f({K}_{m,g})$ and ${K}_{s}\le {K}_{m,g}$. In fact, it has been noted that *K*_*s*_ values for *E. coli* grown with glucose vary significantly in different studies or different growth conditions, and there has been a lack of a satisfactory explanation of why the presumably constant *K*_*s*_ spans across such a wide range^32^. The discrete variable *δ* provides a simple explanation to this long-lasting problem: under strong carbon limitation, the cell benefits from the metabolic strategy where all carbon consumed for energy production is metabolized through respiration; $\delta ={p}_{c}/({p}_{c}+{p}_{E}+{p}_{BM}) < 1$ (Supplementary Eq. (S28)) suggests the relative importance of C, E and BM sectors in dictating the cell’s overall affinity to the growth-controlling substrate. When substrate becomes sufficient which potentially allows the cell to grow at a rate beyond a threshold, it becomes more advantageous for a cell to switch to a metabolic strategy where a significant portion of carbon consumed for energy production is processed through the proteome-efficient acetate pathway. In this case, the C sector touches its lower bound and decouples from E and BM sectors, $\delta ={p}_{c}/{p}_{c}=1$ (Supplementary Eq. (S29)). Essentially, *δ* reflects the metabolic state of a cell. In principle, if a cell possesses a number of metabolic strategies, *δ* would hold the same number of discrete values, each leading to a distinct value of the apparent affinity constant *K*_*s*_. This mechanism could be the underlying reason of the observed large variation in *K*_*s*_ under different culture conditions. Overall, our theoretical model indicates that *K*_*s*_ reflects the combined effect of the local characteristics of the carbon transport system and the overarching interplay between carbon-scavenging and other growth-related functions.

## Discussion

In this work, we modified the classic FBA model (constrained primarily by the mass and energy balance) via embedding the coarse-grained proteome allocation constraints with an intention to reveal the potential mechanistic nature of the empirical growth kinetics proposed by Monod seventy years ago. By doing so, we showed theoretically a non-zero lower bound of the carbon-scavenging sector, which differs from the previous notion that ${\phi }_{C}$ could continuously decrease towards zero as growth rate increases^19^. Under carbon limitation, it seems to be agreeable that ${\phi }_{E}+{\phi }_{BM}$ increases with growth rate^26,27^ and reaches a plateau (defined by ${\phi }_{max}^{o}$) where the acetate overflow occurs so that more proteome could be allocated to biomass synthesis as required for rapid growth^14^. Consequently, the rest of the proteome $({\phi }_{C}+{\phi }_{Q})$ has to decrease and then levels^33^. If ${\phi }_{Q}$ remains constant, taking for example its minimum value $({\phi }_{Qmin})$ as considered in this work for cells striving for maximum growth, the size of the level end of the $({\phi }_{C}+{\phi }_{Q})$ profile will be $({\phi }_{Cmin}+{\phi }_{Qmin})$. We argue that in this level sum, the contribution of ${\phi }_{Cmin}$ is unlikely to be zero, given the significant carbon influx to sustain the high rate of biomass synthesis and energy production under overflow conditions. On the maximum value of ${\phi }_{C}$ $({\phi }_{Cmax})$, our parameter estimation based on data assembled from literature (Supplementary Table S1) determined ${\phi }_{Cmax}=0.37$ for NCM3722 and ${\phi }_{Cmax}=0.77$ for ML308 (${\phi }_{Cmax}\approx {\phi }_{max}^{g}$, determined by extrapolating to zero growth rate, also see Eq. (2) and Fig. 2a,b). These specific values are affected by a number of factors, including particularly (i) the assumed value for ${\phi }_{max}^{o}$ (0.19, previously suggested for *E. coli* NCM3722^14^) and (ii) the value of *K*_*s*_ estimated from cell culture data (Supplementary Text and Supplementary Eqs. (S30) and (S34 and S35)). In reality, the former could be strain-dependent, while the latter is known to be sensitive to growth conditions and culture history^4,32,34^. Therefore, the specific results of parameterization from this work were to support the illustration of the overall modelling approach; their biological significance needs to be interpreted with caution.

In dynamic FBA (DFBA), *v*_*c*_ (uptake rate of growth-limiting carbon source) is generally determined by applying the Michaelis-Menten kinetics for substrate transport^35–39^. An inherent issue with this approach is that the kinetic parameters are normally valid only for a specific substrate uptake pattern, which however may vary depending on the variety of fermentative by-products (e.g. acetate, formate, ethanol or a mixture of them)^10^ or more generally on the physiological state. Therefore, using Michaelis-Menten kinetics alone (with a single set of kinetic parameters) to predict the carbon intake suffers from a very limited scope of applicability – it cannot depict a physiological state that deviates from the state that it was originally fitted to. In this work, this drawback is rectified by introducing the proteome allocation principle, which couples carbon transport with other growth-associated metabolism. Through a fully parameterized ${w}_{c}-[g]$ correlation, we compute the variable *w*_*c*_ given the environmental glucose concentration (as opposed to directly manipulating *w*_*c*_^19^). The computable *w*_*c*_ is involved in the proteome allocation constraints, which allows us to simultaneously predict the glucose uptake rate, specific growth rate and acetate overflow upon varying the environmental glucose level. The coupling of the local metabolic capacity (for carbon transport) and the global regulatory mechanism (on proteomic resource allocation) was shown to be essential in improving the predictive power of FBA models. It should be noted that, while in this work we have adopted a single *K*_*m,g*_ for the carbon transport system, it is known that in *E. coli, K*_*m,g*_ differs from different transporters which could be active either exclusively or in parallel^40,41^. When using a single (aggregated) *K*_*m,g*_ becomes inadequate, a more accurate representation of the carbon transport could be achieved by explicitly modelling individual transporters (see below for further discussion). Besides, the proteome allocation principle could also be revised to investigate proteomic burdens imposed by the expression of heterologous genes^42^.

In this work, we used the core metabolic model of *E. coli* to demonstrate the effectiveness of proteome allocation constraints (Eqs. (16 and 17)) in dictating bacterial growth strategies. However, the proposed modelling framework can be implemented with more detailed genome-scale metabolic models (GEMs)^43–45^ to explore biological insights for *E. coli* and other microorganisms with similar putative mechanisms. The key advance of the GEM over the core model is the extensive information of metabolic reactions originating from genome annotation^46^. Pathways such as alternate carbon metabolism, amino acid metabolism, nucleotide metabolism, cofactor biosynthesis and fatty acid biosynthesis are exclusively modelled in GEMs. To fuse the proteome allocation constraints into a GEM, particular attention is needed for the choice of fluxes underlying *v*_*c*_, *v*_*f*_ and *v*_*r*_, as well as the determination of proteome cost parameters ${w}_{c}^{\ast }$, ${w}_{f}^{\ast }$ and ${w}_{r}^{\ast }$.

We first focus on ${w}_{c}^{\ast }$ and *v*_*c*_. In the core model, glucose is the sole carbon source to support growth and is imported via phosphoenolpyruvate(PEP):pyruvate (PYR) phosphotransferase system (PTS). In this case *v*_*c*_ is related to a one-step transport flux (GLCpts). However in *E. coli* GEM *i*AF1260, glucose can be transported not only via glucose-specific PTS components, but also via alternative routes such as glucose-specific ABC system, glucose:proton symporter or simply via diffusion^43^. In *i*AF1260, glucose import (from extracellular glucose to cytosolic glucose-6-phosphate) is carried out by multiple enzymatic steps, e.g. first via the glucose:proton symporter then through hexokinase, instead of a simplified one-step process. Furthermore, 174 carbon sources were predicted to potentially support growth in *i*AF1260, meaning that growth simulation is not limited to glucose minimal media. The improved details of the metabolic network imply that (a) when modelling cells grown on glucose, *v*_*c*_ may need to be coupled to multiple glucose transporters and (b) when modelling cells grown on alternative or mixed carbon sources, *v*_*c*_ needs to correspond to various carbohydrate transport pathways. Accompanying a more detailed account of substrate transport fluxes, the (normalised) proteome cost parameter for carbon transport ${w}_{c}^{\ast }$ has to be revised. We have shown in this work that ${w}_{c}^{\ast }$ is a function of ${\phi }_{max}^{o}$, ${w}_{c,0}$ and *K*_*m,g*_ (Eqs. 6 and 16) and that *w*_*c*,0_ is a function of ${\phi }_{max}^{o}$ and *K*_*m,g*_ (Supplementary Eq. (S34)); combining the two makes ${w}_{c}^{\ast }$ a function of *K*_*m,g*_ (the Michaelis constant for glucose transport). When multiple transporters exist, as in GEMs, a set of *K*_*m,g*_ (for glucose) or multiple sets of *K*_*m,s*_ (for non-glucose carbon sources) would be required for active transporters in the specific growth conditions. Correspondingly, ${w}_{c}^{\ast }$ will be calculated from the combination of *K*_*m,g*_ for cells grown on a glucose minimal medium. For cells grown on a complex carbon medium, multiple ${w}_{c}^{\ast }$ will be needed (to match distinct transport fluxes) and should be determined by multiple sets of *K*_*m,s*_. In short, integrating the proteome cost constraints into GEM asks for more information of kinetic parameters of carbon transport.

Furthermore, the increased flux variability and potential reactions running in parallel in GEMs may alter the choice of representative fluxes of fermentation and respiration pathways. For example, *v*_*f*_ and *v*_*r*_ in *i*AF1260 can become enolase (ENO) and citrate synthase (CS)^42^. Changed *v*_*f*_ and *v*_*r*_ will subsequently affect the estimation of proteome cost parameters ${w}_{f}^{\ast }$ and ${w}_{r}^{\ast }$ (Supplementary Text).

In conclusion, our model offers a latest mechanistic interpretation of the seventy-year old Monod growth kinetics: the maximum specific growth rate of a microorganism could be governed by the abundance of growth-controlling proteome and the associated proteome cost per unit increase of growth rate. The Monod constant *K*_*s*_ was shown to be quantitatively related to not only the enzymatic affinity for substrate transport *K*_*m,g*_ but also the metabolic state of a cell, which might explain the large variations in reported *K*_*s*_ values. Our analysis also suggests that a microorganism with a lower maintenance cost, higher fraction of growth-controlling proteome and alternative pathways with different enzyme costs likely competes successfully in a changing environment. Finally, the proposed modelling concept eliminates the need for treating substrate intake as an input in FBA simulation, which demonstrates the potential of coupling local and global physiological constrains in predictive modelling for systems biology and synthetic biology.

## Methods

### Modelling proteome allocation in *E. coli*

Following the assumption that the fraction of a proteome sector is proportional to the metabolic flux it processes^26^, Eq. (2) can be rewritten as

14 $${w}_{c}{v}_{c}+{w}_{f}{v}_{f}+{w}_{r}{v}_{r}+b\lambda ={\phi }_{max}^{g}$$

*w*_*i*_ is the enzyme cost per unit flux processed by sector *i* (*i* = *C*, *f or r*). *b* is the proteome cost per unit biomass synthesis flux. *v*_*c*_ is the carbon transport flux. For *E. coli* grown with glucose as the single growth-controlling substrate, *v*_*c*_ is the glucose uptake flux which is normally processed by the phosphoenolpyruvate:sugar phosphotransferase system (PTS)^40,47^. *v*_*f*_ (*v*_*r*_) is the fermentation (respiration) flux; *λ* is the biomass synthesis flux, which in this work is equal to the specific growth rate. From ref. ^28^, the fraction of proteome attainable to energy biogenesis and biomass synthesis is constrained by an upper bound ${\phi }_{max}^{o}$

15 $${w}_{f}^{\ast }{v}_{f}+{w}_{r}^{\ast }{v}_{r}+{b}^{\ast }\lambda \le 1$$

where ${w}_{i}^{\ast }\equiv \frac{{w}_{i}}{{\phi }_{max}^{o}}(\,i=f\,or\,r)$ and ${b}^{\ast }\equiv \frac{b}{{\phi }_{max}^{o}}$ are the (normalised) proteome cost per unit flux. Dividing both sides of Eq. (14) by ${\phi }_{max}^{o}$

16 $${w}_{c}^{\ast }{v}_{c}+{w}_{f}^{\ast }{v}_{f}+{w}_{r}^{\ast }{v}_{r}+{b}^{\ast }\lambda =\frac{{\phi }_{max}^{g}}{{\phi }_{max}^{o}}$$

Substituting Eq. (15) into Eq. (16), under acetate overflow (hence equal sign applied to Eq. (15)

17 $${w}_{c}^{\ast }{v}_{c}=\frac{{\phi }_{max}^{g}}{{\phi }_{max}^{o}}-1$$

It follows that

18 $${\phi }_{Cmin}={\phi }_{max}^{g}-{\phi }_{max}^{o}$$

${\phi }_{Cmin}$ is the minimum proteome fraction allocated to the carbon-scavenging sector (occurring during acetate overflow). ${\phi }_{max}^{g} > {\phi }_{max}^{o}$ (due to the inclusion of the C sector). Equations (16 and 17) are the proteome allocation constraints adopted in model simulations (see below).

### Deriving the hyperbolic $\lambda -[g]$ correlation

Starting from the Michaelis-Menten kinetics together with the proteome allocation embedded FBA model presented in this work, we have derived a hyperbolic $\lambda -[g]$ correlation that is comparable to the Monod equation and reveals the potential biological mechanisms that underpin the phenomenological Monod parameters. Detailed mathematical derivation is provided in the Supplementary Text.

### Determination of GAM and ATPM

In FBA models, the energy consumption is dictated primarily via growth-associated maintenance (GAM) and non-growth associated maintenance (ATPM), both of which are difficult to quantify accurately^43^. GAM and ATPM could also vary between different species and strains (BiGG database, http://bigg.ucsd.edu/). Therefore we modified the default values of GAM and ATPM according to strain-specific energetic data (Supplementary Table S2). To do so we associated GAM with the molar growth yield (namely the true growth yield *Y*_*G*_^48^). ATPM is linked to the maintenance coefficient (*m*), which corresponds to the rate of substrate uptake extrapolated to zero growth rate.

### Model simulation

The following linear programing problem was solved in FBA simulations of the growth of *E. coli* under different extracellular glucose concentrations. FBA was performed based on the core *E. coli* metabolic model^49^.

19 $$\begin{array}{c}maximise\,\lambda \\ subject\,to\,Sv=0\\ \,lb\le {v}_{i}\le ub\\ \,{w}_{f}^{\ast }{v}_{f}+{w}_{r}^{\ast }{v}_{r}+{b}^{\ast }\lambda \le 1\\ \,{w}_{c}^{\ast }{v}_{c}+({w}_{f}^{\ast }{v}_{f}+{w}_{r}^{\ast }{v}_{r}+{b}^{\ast }\lambda )={\phi }_{max}^{g}/{\phi }_{max}^{o}\\ \,{w}_{c}^{\ast }{v}_{c}={\phi }_{max}^{g}/{\phi }_{max}^{o}-1\,(if\,\lambda \ge {\lambda }_{ac})\end{array}$$

*v*_*c*_ is represented by the first enzymatic step of glycolysis, GLCpts, *v*_*f*_ is the acetate synthesis reaction ACKr, *v*_*r*_ adopts the first reaction after biomass withdrawal in TCA cycle, AKGDH. *λ* is growth rate and refers to the biomass reaction. *λ*_*ac*_ is the threshold growth rate, above which acetate overflow occurs. The determination of model parameters (${w}_{c}^{\ast }$, ${w}_{f}^{\ast }$, ${w}_{r}^{\ast }$, ${b}^{\ast }$, ${\phi }_{max}^{o}$ and ${\phi }_{max}^{g}$) is detailed in the Supplementary Text. The lower bound of glucose and oxygen exchange fluxes were set to −1000 to avoid artificial control and to allow prediction of their uptake flux. Enzymatic reactions ICL, MALS, FRD7 were switched off under aerobic-glucose conditions according to^43^. Irrelevant exchange fluxes around the pyruvate node (EX_pyr(e), EX_lac(e), EX_acald(e), EX_etoh(e)) and the α-ketoglutarate node (EX_glu_L(e) and EX_akg(e)) were closed. Maximum carbon flow to the PP pathway was limited to 67% and 42% of the overall carbon intake for ML308 and NCM3722, respectively according to ref. ^28^. Extracellular glucose concentration was adopted as sole model input (varies from 0.1–75 *μM* for NCM3722 and 0.1–26 *μM* for ML308). The optimisation model was converted to an MILP problem and solved using the Gurobi optimizer (see Supplementary Text for details). FBA was solved via COBRA Toolbox^50^. All simulations were performed in MATLAB R2016a. Experimental data used to compare with the simulation results were obtained from Fig. 1 of ref. ^14^, Fig. 3B of ref. ^19^ and SI Fig. 1 of ref. ^51^ for NCM3722 and from Table 7 of ref. ^52^, Fig. 3b of ref. ^53^ and Fig. 1 of ref. ^34^ for ML308.

## Supplementary information

Supplementary Information.

## Data Availability

All model equations and parameter values are included in the main text or in the Supplementary Information. The MATLAB code for running simulations and the generated datasets in this study are available upon request from the corresponding author.
