# Supplementary material for: Bridging substrate intake kinetics and bacterial growth phenotypes with flux balance analysis incorporating proteome allocation
Source: Sci Rep. 2020 Mar 9;10:4283. doi: 10.1038/s41598-020-61174-0 (PMC7062752; doi:10.1038/s41598-020-61174-0)
Supplement: Supplementary file 1 — Supplementary Information. [file 41598_2020_61174_MOESM1_ESM.pdf]

Supplementary Information to

Bridging substrate intake kinetics and bacterial growth phenotypes with flux balance analysis incorporating proteome allocation

Hong Zeng [1], Aidong Yang [1*]

[1]Department of Engineering Science, University of Oxford, Parks Road, Oxford, OX1 3PJ, UK

*Correspondence: aidong.yang@eng.ox.ac.uk

Supplementary Text

Deriving the hyperbolic $\boldsymbol{\lambda - [g]}$ correlation

From the Michaelis-Menten kinetics for carbon transport, the following correlation between w_c and the environmental substrate level has previously been proposed [1]

$$w_c = w_{c,0}\left(1 + \frac{K_{m,g}}{[g]}\right) \quad \text{(S1)}$$

where $w_{c,0}$ is a constant that reflects the lowest proteome cost per unit carbon transport flux. $[g]$ is the extracellular glucose concentration, where glucose is taken as a representative substrate. $K_{m,g}$ is the Michaelis constant for glucose transport. We assumed a linear correlation between growth rate and glucose uptake rate according to ref. [2]

$$v_c = \begin{cases} k_1\lambda + b_1 \ (\lambda < \lambda_{ac}) \\ k_2\lambda + b_2 \ (\lambda \geq \lambda_{ac}) \end{cases} \quad \text{(S2)}$$

where k_1, b_1, k_2, b_2 are linear coefficients. Further based on the observed linear dependence of a proteome sector on growth rate [3], we obtained the following for the E sector and the BM sector:

$$\frac{\phi_E + \phi_{BM}}{\phi_{max}^o} = w_f^* v_f + w_r^* v_r + b^*\lambda = \begin{cases} k_3\lambda + b_3 < 1 \ (\lambda < \lambda_{ac}) \\ 1 \qquad\qquad\quad (\lambda \geq \lambda_{ac}) \end{cases} \quad \text{(S3)}$$

where k_3 and b_3 are linear coefficients. Substituting equations (S2-S3) into equation (16) of the main text (i.e. $w_c^* v_c + w_f^* v_f + w_r^* v_r + b^*\lambda = \frac{\phi_{max}^g}{\phi_{max}^o}$), for non-overflow growth ($\lambda < \lambda_{ac}$):

$$w_c^* = \frac{\frac{\phi_{max}^g}{\phi_{max}^o} - (k_3\lambda + b_3)}{(k_1\lambda + b_1)} \quad \text{(S4)}$$

Comparing equation (S4) with equation (S1) (noted that $w_c = w_c^* \phi_{max}^o$) gives

$$\left(\frac{\frac{\phi_{max}^g}{\phi_{max}^o} - (k_3\lambda + b_3)}{k_1\lambda + b_1}\right)\phi_{max}^o = w_{c,0}\left(1 + \frac{K_{m,g}}{[g]}\right) \quad \text{(S5)}$$

Rearranging equation (S5) to represent λ as a function of $[g]$:

$$\lambda = \frac{(\phi_{max}^g - b_3\phi_{max}^o - w_{c,0}b_1)[g] - w_{c,0}b_1K_{m,g}}{(w_{c,0}k_1 + k_3\phi_{max}^o)[g] + w_{c,0}k_1K_{m,g}} \quad \text{(S6)}$$

Equation (S6) can be re-arranged to a hyperbolic form (equation (S30)). For example, this can be done by first dividing the numerator and the denominator of equation (S6) by $w_{c,0}k_1$

$$\lambda = \frac{\left(\frac{\phi^{g}_{max}}{w_{c,0}k_1} - \frac{\phi^{o}_{max}b_3}{w_{c,0}k_1} - \frac{b_1}{k_1}\right)[g] - \frac{b_1}{k_1}K_{m,g}}{\left(\frac{\phi^{o}_{max}k_3}{w_{c,0}k_1} + 1\right)[g] + K_{m,g}} \tag{S7}$$

Introducing a facilitating term $\left(+\frac{b_1}{k_1}\frac{\phi^{o}_{max}k_3}{w_{c,0}k_1}[g] - \frac{b_1}{k_1}\frac{\phi^{o}_{max}k_3}{w_{c,0}k_1}[g]\right)$ to the numerator of equation (S7)

$$\lambda = \frac{\left(\frac{\phi^{g}_{max} - \phi^{o}_{max}b_3}{w_{c,0}k_1}\right)[g] - \frac{b_1}{k_1}[g] - \frac{b_1}{k_1}K_{m,g} + \frac{b_1}{k_1}\frac{\phi^{o}_{max}k_3}{w_{c,0}k_1}[g] - \frac{b_1}{k_1}\frac{\phi^{o}_{max}k_3}{w_{c,0}k_1}[g]}{\left(\frac{\phi^{o}_{max}k_3}{w_{c,0}k_1} + 1\right)[g] + K_{m,g}} \tag{S8}$$

Lumping the $\frac{b_1}{k_1}$ related terms together

$$\lambda = \frac{\left(\frac{\phi^{g}_{max} - \phi^{o}_{max}b_3}{w_{c,0}k_1} + \frac{b_1}{k_1}\frac{\phi^{o}_{max}k_3}{w_{c,0}k_1}\right)[g] - \frac{b_1}{k_1}\left(\left(\frac{\phi^{o}_{max}k_3}{w_{c,0}k_1} + 1\right)[g] + K_{m,g}\right)}{\left(\frac{\phi^{o}_{max}k_3}{w_{c,0}k_1} + 1\right)[g] + K_{m,g}} \tag{S9}$$

Finally, rearranging equation (S9) to obtain a hyperbolic $\lambda - [g]$ correlation

$$\lambda = \frac{\phi^{g}_{max} - \phi^{o}_{max}\left(b_3 - \frac{b_1}{k_1}k_3\right)}{w_{c,0}k_1 + \phi^{o}_{max}k_3}\frac{[g]}{[g] + \frac{w_{c,0}k_1}{w_{c,0}k_1 + \phi^{o}_{max}k_3}K_{m,g}} - \frac{b_1}{k_1} \tag{S10}$$

Similarly for overflow growth ($\lambda \geq \lambda_{ac}$), substituting equations (S2-S3) into equation (16) of the main text

$$w_c^* = \frac{\frac{\phi^{g}_{max}}{\phi^{o}_{max}} - 1}{k_2\lambda + b_2} \tag{S11}$$

Comparing equation (S11) with equation (S1)

$$\left(\frac{\frac{\phi^{g}_{max}}{\phi^{o}_{max}} - 1}{k_2\lambda + b_2}\right)\phi^{o}_{max} = w_{c,0}\left(1 + \frac{K_{m,g}}{[g]}\right) \tag{S12}$$

Rearrange equation (S12) to represent λ as a function of $[g]$

$$\lambda = \frac{\phi^{g}_{max} - \phi^{o}_{max}}{w_{c,0}k_2}\frac{[g]}{[g] + K_{m,g}} - \frac{b_2}{k_2} \tag{S13}$$

Comparing equations (S10) and (S13) with the Monod equation, i.e. $\lambda = \lambda_{max}\frac{[g]}{[g]+K_s}$, we obtain the following matches (also see equation (S30)):

$$\lambda_{max} = \begin{cases} \frac{\phi^{g}_{max} - (\phi^{o}_{max}b_3 + w_{c,0}b_1)}{w_{c,0}k_1 + \phi^{o}_{max}k_3} & (\lambda < \lambda_{ac}) \\ \frac{\phi_{c,min} - w_{c,0}b_2}{w_{c,0}k_2} & (\lambda \geq \lambda_{ac}) \end{cases} \tag{S14}$$

and

$$K_s = \begin{cases} \frac{w_{c,0}k_1}{w_{c,0}k_1 + \phi^{o}_{max}k_3}K_{m,g} & (\lambda < \lambda_{ac}) \\ K_{m,g} & (\lambda \geq \lambda_{ac}) \end{cases} \tag{S15}$$

Below we show how to resolve the physical links between the Monod kinetic parameters (λ_{max} and K_s) and cell's physiological state through analyzing the biological meaning of the denominator and the numerator of equations (S14-S15).

Resolving λ_{max}

Starting with the non-overflow scenario ($\lambda < \lambda_{ac}$), we first focus on the numerator of the derived expression of λ_{max} (equation (S14)). b_3 is the proteome fraction accounting for non-growth maintenance in E and BM sectors (normalised by ϕ^o_{max}; *cf.* equation (S3)), therefore

$$\phi^o_{max} b_3 = (\phi_E + \phi_{BM})_{atpm} \tag{S16}$$

where the subscript $atpm$ denotes the proteome fraction occupied by non-growth-associated maintenance. Furthermore, b_1 is the rate of carbon intake for maintenance purposes, therefore the product of b_1 and $w_{c,0}$ (lowest enzyme cost per unit carbon influx, equation (S1)) quantifies the (lowest) portion of the C sector proteome that is occupied for maintenance.

$$w_{c,0} b_1 = \phi_{C,atpm} \tag{S17}$$

Combining equations (S16) and (S17), the numerator of equation (S14) (the branch for $\lambda < \lambda_{ac}$) can be expressed as

$$\phi^g_{max} - \left(\phi_{E,atpm} + \phi_{BM,atpm} + \phi_{C,atpm}\right) = \phi^g_{max} - \sum_i \phi_{i,atpm} \tag{S18}$$

where i represents a proteome sector (C, E, or BM). Moving on to the denominator, $w_{c,0} k_1$ can be coupled to the carbon influx (via equation (S2)), thus representing the (lowest) demand of C sector proteome per unit increase of growth rate, denoted by p_c

$$w_{c,0} k_1 = w_{c,0} \frac{dv_c}{d\lambda} = \left(\frac{d\phi_C}{d\lambda}\right)_{w_c = w_{c,0}} = p_c \tag{S19}$$

Similarly $\phi^o_{max} k_3$ is the summation of the proteome cost (per unit increase of growth rate) for E and BM sectors (*cf.* equation (S3))

$$\phi^o_{max} k_3 = \frac{d\phi_E}{d\lambda} + \frac{d\phi_{BM}}{d\lambda} = p_E + p_{BM} \tag{S20}$$

Combining equations (S19) and (S20), the denominator $\left(w_{c,0} k_1 + \phi^o_{max} k_3\right)$ can be expressed as $p_c + p_E + p_{BM} \equiv \sum_i p_i$, where i represents a proteome sector (C, E, or BM). Summarising, the maximum specific growth rate for non-overflow growth can be quantified as

$$\lambda_{max} = \frac{\phi^g_{max} - \sum_i \phi_{i,atpm}}{\sum_i p_i} \quad (\lambda < \lambda_{ac}) \tag{S21}$$

During acetate overflow ($\lambda \geq \lambda_{ac}$), the numerator of the derived expression of λ_{max} in equation (S14) can be viewed as the (adjusted) proteome abundance of C sector, denoted as ϕ'_c below, which is made proportional to λ by applying an offset term $w_{c,0} b_2$ (Fig. S2).

$$\phi_{c,min} - w_{c,0} b_2 = \phi'_c \tag{S22}$$

On the other hand, the denominator $w_{c,0} k_2$ is comparable with equation (S19), which represents the (lowest) demand of C sector proteome per unit increase of growth rate (p_c) under overflow conditions, i.e.

$$w_{c,0} k_2 = p_c \tag{S23}$$

Thus, during acetate overflow λ_{max} can be interpreted as

$$\lambda_{max} = \frac{\phi'_c}{p_c} \quad (\lambda \geq \lambda_{ac}) \tag{S24}$$

which suggests that under such condition λ_{max} could be dictated only by the characteristics of the carbon-scavenging sector. Equations (S21) and (S24) can be generalised as

$$\lambda_{max} = \frac{\phi_{growth}}{p_{growth}} \tag{S25}$$

where ϕ_{growth} represents the fraction of the growth-controlling proteome, and p_{growth} denotes the proteome cost per unit increase of growth rate.

Resolving K_s

Re-writing equation (S15) to

$$K_s = \delta K_{m,g} \tag{S26}$$

$$\delta = \begin{cases} \frac{w_{c,0}k_1}{w_{c,0}k_1+\phi_{max}^o k_3} < 1 \ (\lambda < \lambda_{ac}) \\ 1 \ \ (\lambda \geq \lambda_{ac}) \end{cases} \tag{S27}$$

We showed that the Monod constant K_s is linked to the Michaelis constant for carbon transport $K_{m,g}$ through a proportional factor δ that possesses discrete values. Given the above analysis for λ_{max}, the biological meaning of the expression of δ (for $\lambda < \lambda_{ac}$) could be shown through

$$\delta = \frac{w_{c,0}k_1}{w_{c,0}k_1+\phi_{max}^o k_3} = \frac{p_C}{p_C+p_E+p_{BM}} < 1 \tag{S28}$$

For the overflow scenario ($\lambda \geq \lambda_{ac}$), $\delta = 1$ actually derives from

$$\delta = \frac{w_{c,0}k_2}{w_{c,0}k_2} = \frac{p_C}{p_C} = 1 \tag{S29}$$

Overall, the discrete feature of the proportional factor δ reflects the switch in a cell's metabolic state (due to global proteome allocation).

Parameterization of $w_c - [g]$ correlation (determining ϕ_{max}^g and $w_{c,0}$)

Equations (S10) and (S13) can be written in a generic form

$$\lambda = A\frac{[g]}{[g]+B} - C \tag{S30}$$

In 1965, Pirt explicitly pointed out that in bacteria some substrate is consumed for functions other than the synthesis of new cells [4]. As mentioned in the main text, the original Monod equation does not recognize such non-growth maintenance. Throughout the years researchers have modified the classic Monod equation by expressing the maintenance as the maintenance rate [4–6], maintenance coefficient [4,7,8] or the threshold substrate concertation (S_{min}) [9–12]. In this work, term C denotes the cellular maintenance. Consequently λ_{max} could be computed by $A - C$ and K_s is equivalent to B. If maintenance is negligible, $C = 0$ (thus $b_1 = b_3 = 0$); equation (S30) reduces to the original Monod equation.

For *E. coli*, the experimentally measured $\lambda - [g]$ profile has been extensively reported in literature. With a proper set of data, constants A, B and C can readily be determined by fitting the growth data to equation (S30). It is worth nothing that the shape of a $\lambda - [g]$ curve is generally determined by the slow-growing region where growth rate changes dramatically with the substrate concentration [13]. Therefore, we chose to fit the growth data to the non-overflow $\lambda - [g]$ correlation (equation (S10)).

$$A = \frac{\phi_{max}^g - \phi_{max}^o\left(b_3 - \frac{b_1}{k_1}k_3\right)}{w_{c,0}k_1+\phi_{max}^o k_3} \tag{S31}$$

$$B = \frac{w_{c,0}k_1}{w_{c,0}k_1+\phi_{max}^o k_3}K_{m,g} \tag{S32}$$

$$C = \frac{b_1}{k_1} \tag{S33}$$

$k_1 - k_3$ and $b_1 - b_3$ (Table S3) are the known parameters that are dictated by the stoichiometry of the metabolic model. ϕ_{max}^o was previously reported to be 0.19 for *E. coli* NCM3722 [14]. We did not find similar data for *E. coli* ML308; therefore we set $\phi_{max}^o = 0.19$ for both strains. The values of A and B were fitted to the experimental data ($\lambda - [g]$ profile) through fixing $C = \frac{b_1}{k_1}$. The unknown parameters are: ϕ_{max}^g, $w_{c,0}$ and $K_{m,g}$,

which are located in equations (S31) and (S32). As we were not able to determine three unknowns with two equations, the value of one unknown needed to be specified to determine the rest. We chose $K_{m,g}$ to be specified as its value has been extensively reported in literature (in a range of 4 – 20 μM [15–22]). Given a known $K_{m,g}$ we could proceed to determine the other parameters. It is worth noting that proteome fraction ϕ_{max}^{g} has to be less than one, which further constrains the feasible range of $K_{m,g}$ (via equation (S35)).

$$w_{c,0} = \frac{\phi_{max}^{o} B}{\frac{k_1}{k_3}(K_{m,g} - B)} \quad \text{(S34)}$$

$$\phi_{max}^{g} = \phi_{max}^{o}\left(k_3 \frac{A K_{m,g}}{K_{m,g} - B} - k_3 C + b_3\right) < 1 \quad \text{(S35)}$$

For strain NCM3722, we fixed $C = 0$ (due to negligible maintenance) and fitted the experimentally determined $\lambda - [g]$ profile of the same strain (SI figure 1 of ref.[23]) to equation (S30), which gives $A = 1$ h^{-1} and $B = 5\ \mu M$. $K_{m,g}$ needs to be higher than 6.6 μM to allow $\phi_{max}^{g} < 1$. We set $K_{m,g} = 15\ \mu M$ according to refs. [20,21], which satisfactorily describes the measured $\lambda - [g]$ data (Fig. S3a). Similarly for strain ML308, we fixed $C = \frac{b_1}{k_1} = 0.0036$. The experimental growth data (figure 1 of ref.[24]) leads to $A = 1.2$ h^{-1} and $B = 12.4\ \mu M$. In this case $K_{m,g}$ has to be higher than 17.6 μM to ensure $\phi_{max}^{g} < 1$. We set $K_{m,g}$ to 20 μM as it gives the best fit to the experimental data (Figs. S3b and S3c). Substituting A, B, C and $K_{m,g}$ values into equations (S34-S35), ϕ_{max}^{g} and $w_{c,0}$ could be computed (Table S1). Inserting the calculated value of $w_{c,0}$ and the associated $K_{m,g}$ specified above into equation (S1), we were able to compute w_c given glucose concentration:

$$w_{c,NCM3722} = 0.0097(1 + 15/[g]) \quad \text{(S36)}$$

$$w_{c,ml308} = 0.0381(1 + 20/[g]) \quad \text{(S37)}$$

where [g] is in μM. The calculated values of ϕ_{max}^{g} were adopted in the proteome allocation constraints to simulate the cell growth (equation (19) of the main text).

Determining the proteome cost parameters

The model comprises four proteome cost parameters denoting the proteome cost per unit flux (see equation (16) of the main text): w_c^*, w_f^*, w_r^* and b^*. The variable w_c^* could be calculated via dividing equations (S36-S37) by ϕ_{max}^{o}. The rest of the proteome cost parameters (i.e. w_f^*, w_r^* and b^*) could be determined via the experimentally determined acetate production rate against growth rate profile (referred to as the acetate line) and the respiration flux against growth rate profile (referred to as the respiration line). As no directly measured respiration line were reported along with the acetate line, we fixed the growth rate and acetate excretion flux according to the acetate line and performed FBA simulation with minimizing glucose uptake rate as the objective function to obtain an estimated respiration line. The slope and intercept of the acetate line and the respiration line were used to determine w_f^*, w_r^* and b^* (Table S1) following the approach detailed in ref. [25].

Comments on the approach for determining model parameters

The model proposed in this work comprises five critical parameters: w_c, w_f, w_r, b, ϕ_{max}^{o} and ϕ_{max}^{g} (or equivalently, w_c^*, w_f^*, w_r^*, b^* and $\phi_{max}^{g}/\phi_{max}^{o}$), among which w_c is a variable (depending on extracellular glucose concentration) while the rest are constants. The determination of w_c relies on two parameters ($w_{c,0}$ and $K_{m,g}$) involved in the $w_c - [g]$ correlation (equation (S1)). $K_{m,g}$ is the Michaelis constant for carbon transport, which has been extensively reported in literatures. From Mori *et al.*'s original work [1], $w_{c,0}$ combines several physiological parameters, including cell dry weight, molecular weight of carbon transport enzymes, mass of total proteins in a cell, enzyme turnover rate and the mass fraction of carbon transport enzymes. It is unlikely to directly compute $w_{c,0}$ unless all these parameters (including $K_{m,g}$) were measured in a consistent set of experiments. To circumvent this difficulty, in this work we have essentially treated $w_{c,0}$ as a phenomenological parameter and determined it using bioreactor-level growth data, i.e. the $\lambda - [g]$ profile. On the other hand, previous work has shown that a set of w_f, w_r and b could be determined if we know the

information of measured acetate excretion and (measured or deduced) respiration flux [25]. Finally ϕ_{max}^{o} and ϕ_{max}^{g}, by their definition, could be obtained directly from quantitative proteome analysis. If the proteome data is not available, ϕ_{max}^{g} could be estimated via the experimentally determined $\lambda - [g]$ profile, as done in this work. One can see that the maximum value of ϕ_C (ϕ_{Cmax}) is directly associated to ϕ_{max}^{g} (equation (2), Figs. 2a and 2b of the main text), which in turn is affected by ϕ_{max}^{o} and K_s (i.e. B) in equations (S34-S35). In fact, it was primarily the much larger K_s of ML308 than that of NCM3722 which led to a much larger ϕ_{Cmax} (and ϕ_{max}^{g}) of the former. K_s is known to be sensitive to growth conditions and culture history [10,24,26], hence its estimated value can be very much attached to the particular set of cell culture data used. Therefore, the parameterization results obtained as such need to be treated with caution.

Formulating the mixed integer linear programing (MILP) problem

The constraint $w_c^* v_c = \phi_{max}^{g}/\phi_{max}^{o} - 1\ (\lambda \geq \lambda_{ac})$ in equation (19) of the main text is derived from equation (17), meaning that $w_c^* v_c$ equals to a constant, $\phi_{max}^{g}/\phi_{max}^{o} - 1$, at $v_f > 0$. It ensures that the acetate production (i.e. the activation of the fermentation pathway) occurs simultaneously with the activation of the equal sign of the proteome constraint for overflow metabolism (equation (15) of the main text), i.e. $v_f > 0$ at $w_f^* v_f + w_r^* v_r + b^* \lambda = 1$. To avoid introducing a conditional constraint in the FBA model, this constraint can be re-expressed as

$$z v_f = 0\ (z = 0\ or\ 1) \tag{S38}$$

$$[w_c^* v_c - \theta](1 - z) = 0 \tag{S39}$$

where $\theta = \phi_{max}^{g}/\phi_{max}^{o} - 1$. To further avoid solving a non-linear optimisation problem, we converted the bilinear term $z v_f$ in equation (S38) to:

$$\begin{gathered} y_1 = z v_f \\ y_1 \leq z v_{f,max} \\ y_1 \leq v_f \\ y_1 \geq v_f - v_{f,max}(1 - z) \\ y_1 \geq 0 \end{gathered}$$

Similarly, the bilinear term $z v_c$ in equation (S39) was converted to

$$\begin{gathered} y_2 = z v_c \\ y_2 \leq z v_{c,max} \\ y_2 \leq v_c \\ y_2 \geq v_c - v_{c,max}(1 - z) \\ y_2 \geq 0 \end{gathered}$$

where y_1, y_2 are continuous variables, z is a binary variable ($z = 0\ or\ 1$). $v_{f,max} = 1000$, $v_{c,max} = 1000$. Overall we have the following MILP problem, which is mathematically equivalent to the original problem (equation (19) of the main text):

$$\begin{array}{lll} maximise\ \lambda & & \\ subject\ to & Sv = 0 & y_1 \leq v_f \\ & lb \leq v_i \leq ub & y_1 \geq v_f - v_{f,max}(1 - z) \\ & w_f^* v_f + w_r^* v_r + b^* \lambda \leq 1 & y_2 \leq z v_{c,max} \\ & w_c^* v_c + w_f^* v_f + w_r^* v_r + b^* \lambda = \phi_{max}^{g}/\phi_{max}^{o} & y_2 \leq v_c \\ & y_1 = 0 & y_2 \geq v_c - v_{c,max}(1 - z) \\ & w_c^* v_c - \theta + \theta z - w_c^* y_2 = 0 & y_1 \geq 0 \\ & y_1 \leq z v_{f,max} & y_2 \geq 0 \\ & z = 0\ or\ 1 & \end{array}$$

Supplementary Figures and Tables

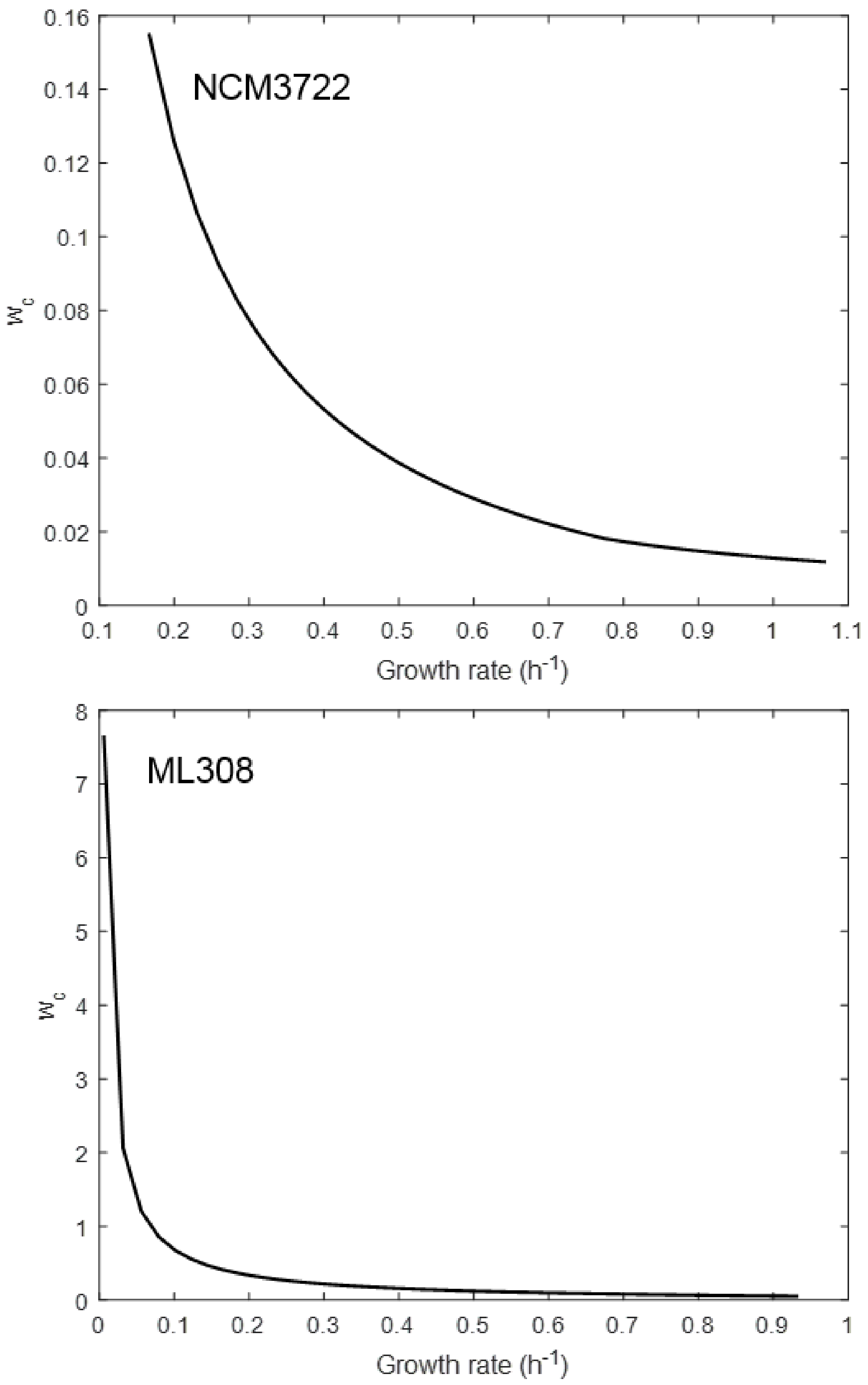

Figure S1. Variation of w_c against simulated specific growth rate for NCM3722 and ML308.

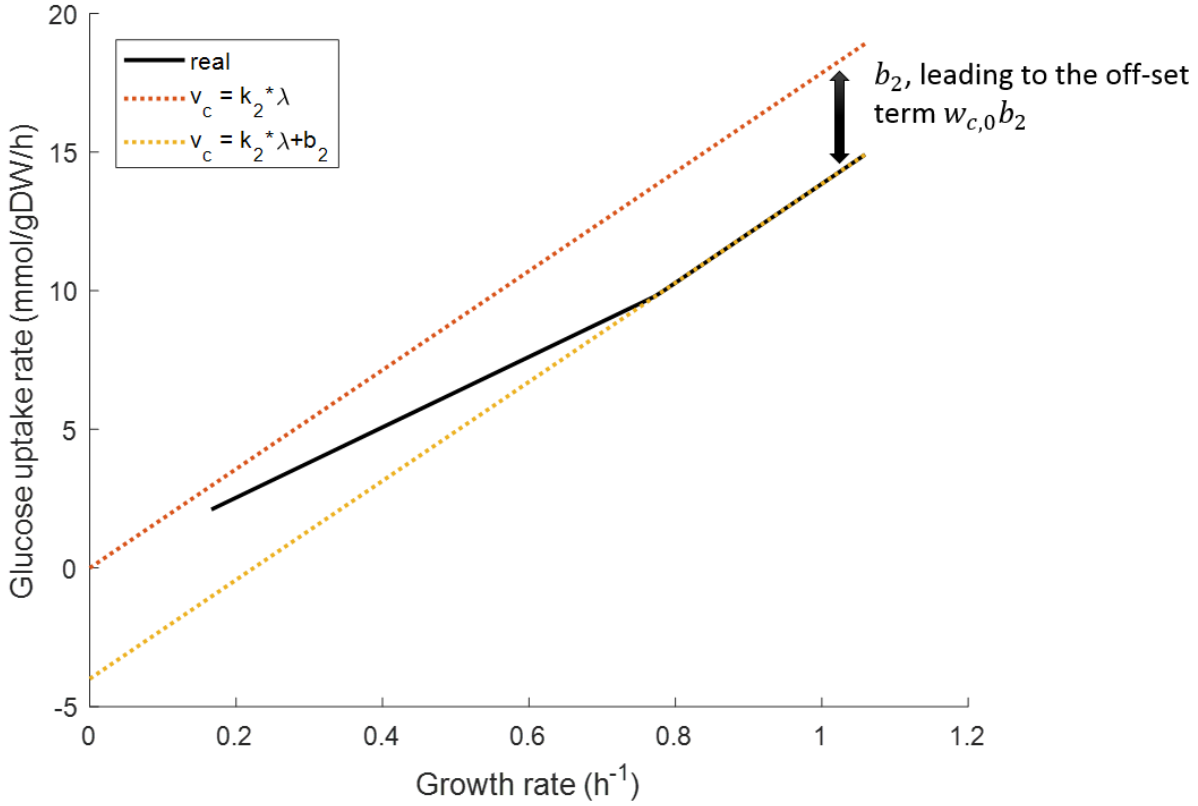

Figure S2. Difference between the real, proportional and linear correlation between $v_c - \lambda$ pair, which results in an offset term $w_{c,0}b_2$ in equation (S22).

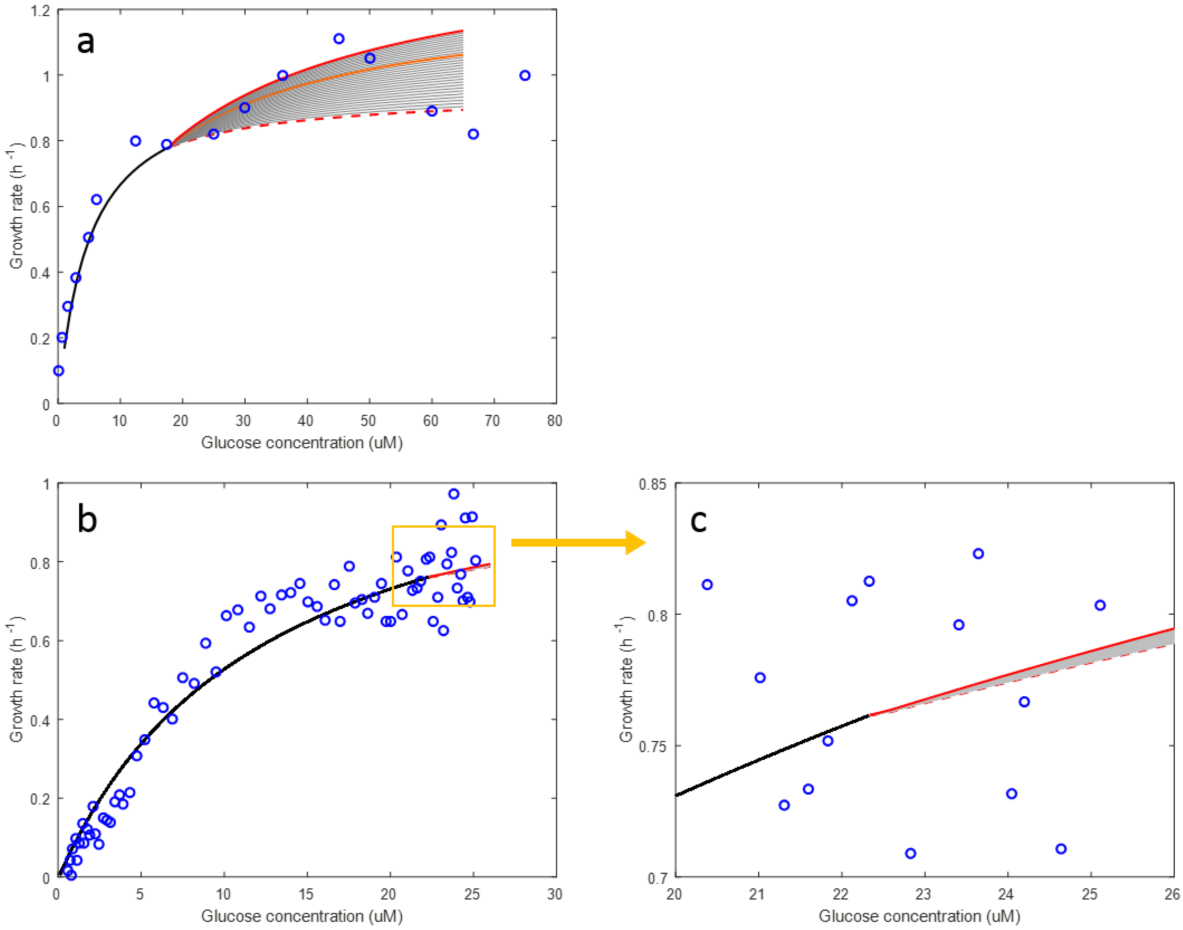

Figure S3. Impact of varying $K_{m,g}$ on predicted $\lambda - [g]$ profile. **(a)** Comparison between the simulated (upon varying $K_{m,g}$) and measured $\lambda - [g]$ profile for NCM3722. Blue circles are the experimental data obtained from SI figure 1 of ref. [23]. For $\lambda < \lambda_{ac}$ (~0.8) simulated $\lambda - [g]$ correlation is described by equation (S10). For $\lambda \geq \lambda_{ac}$, the simulation result is governed by equation (S13). In the overflow region, the variation of $K_{m,g}$ results in different model predictions. Grey area: $K_{m,g}$ varies between $5.5 - 20\ \mu M$. Red dashed line: $K_{m,g} = 5.5\ \mu M$, orange solid line: $K_{m,g} = 15\ \mu M$ (best fit for NCM3722), red solid curve: $K_{m,g} = 20\ \mu M$. **(b-c)** Comparison between the simulated (upon varying $K_{m,g}$) and measured $\lambda - [g]$ profile for ML308. Blue circles are the experimental data obtained from figure 1 of ref. [24]. For $\lambda < \lambda_{ac}$ (~0.76) simulated $\lambda - [g]$ correlation is described by equation (S10). For $\lambda \geq \lambda_{ac}$, the simulation result is governed by equation (S13). In the overflow region, the variation of $K_{m,g}$ results in different model predictions. Grey area: $K_{m,g}$ varies between $15 - 20\ \mu M$. Red dashed line: $K_{m,g} = 15\ \mu M$, red solid curve: $K_{m,g} = 20\ \mu M$ (best fit for ML308).

Table S1. Parameters involved in the proteome allocation constraints.

**Parameter (Units)**	**Description**	**Value**		**Source**
		ML308	NCM3722	
b^* (gDW h/mmol)	Normalized proteome cost per unit biomass synthesis flux	0.1	0.1	
k_f (mmol/gDW)	Slope of the acetate line	22.38	19.28	
k_r (mmol/gDW)	Slope of the respiration line	-9.80	-2.84	
$v_{f,0}$ (mmol/gDW/h)	y-axis intercept of the acetate line	-16.17	-14.42	
$v_{r,0}$ (mmol/gDW/h)	y-axis intercept of the respiration line	9.76	8.65	This work
$w_{c,0}$ (gDW h/mmol)	Lowest enzyme cost per unit carbon transport flux	0.0381	0.0097	
w_f^* (gDW h/mmol)	Normalized enzyme cost per unit fermentation flux	0.1470	0.0157	
w_r^* (gDW h/mmol)	Normalized enzyme cost per unit respiration flux	0.3458	0.1417	
ϕ_{max}^{g} (-)	Maximal proteome fraction attainable for C, E and BM sectors	0.7728	0.3680	
ϕ_{max}^{o} (-)	Maximal proteome fraction attainable for BM and E sectors	0.19		[14]

Note:

k_f and $v_{f,0}$ were determined from the experimentally determined acetate line. For ML308, data were obtained from table 7 of ref. [27]. For NCM3722, data were obtained from figure 1 of ref. [14].

k_r and $v_{r,0}$ were obtained from FBA simulated respiration line.

Table S2. Reported values of the maintenance coefficient and the molar growth yield and fitted energy parameters for different *E. coli* strains.

**Strain**	$\boldsymbol{m}$ **(mmol/gDW/h)**	$\boldsymbol{Y_G}$ **(gDW/mol glc)**	**ATPM (mmol /gDW/h)**	**GAM (mmol /gDW)**	**Source**
ML308	0.038[a]	95.0[a]	0.65[b]	52.7[b]	[7]
NCM3722	0[a]	78.8[b]	0[a]	91.4[a]	[14]

[a.] Directly extracted from literature.
[b.] Calculated in this work.

Note:

m is the maintenance coefficient, which denotes the rate of substrate consumed for non-growth maintenance.

$Y_G = \frac{\Delta x}{(\Delta s)_G} = \frac{\Delta \lambda}{\Delta v_C}$ is the molar growth yield (or true growth yield), which involves the production of a certain amount of biomass Δx and the consumption of growth-associated substrate $(\Delta s)_G$ [4].

1 OD = 0.5 gDW was applied for the conversion of GAM for NCM3722 [1].

Table S3. Parameters involved in determining the $w_c - [g]$ correlation.

Parameter (Units)	Description	Value		Source
		ML308	NCM3722	
b_1 (mmol/gDW/h)	Rate of glucose uptake for non-growth maintenance	3.78E-2	0	[7,14]
b_2 (mmol/gDW/h)	y-axis intercept of $v_c - \lambda$ line for overflow growth	-5.022	-3.998	This work
b_3 (-)	E and BM sector proteome fraction for non-growth maintenance	2.31E-2	0	This work
k_1 (mmol/gDW)	Inverse of molar growth yield for non-overflow growth	10.53	12.69	[7,14]
k_2 (mmol/gDW)	Inverse of molar growth yield for overflow growth	17.19	17.85	This work
k_3 (h)	Slope of $\phi_E + \phi_{BM}$ against λ line	1.287	1.291	This work

References:

1. Mori, M., Hwa, T., Martin, O. C., De Martino, A. & Marinari, E. Constrained Allocation Flux Balance Analysis. *PLoS Comput. Biol.* **12**, (2016).
2. Varma, A., Boesch, B. W. & Palsson, B. Ø. Stoichiometric interpretation of Escherichia coli glucose catabolism under various oxygenation rates. *Appl. Environ. Microbiol.* **59**, 2465–2473 (1993).
3. Hui, S. *et al.* Quantitative proteomic analysis reveals a simple strategy of global resource allocation in bacteria. *Mol. Syst. Biol.* **11**, (2015).
4. Pirt, S. J. The maintenance energy of bacteria in growing cultures. *Proc. R. Soc. Lond. B* **163**, 224–231 (1965).
5. Marr, A. G., Nilson, E. H. & Clark, D. J. THE MAINTENANCE REQUIREMENT OF ESCHERICHIA COLI. *Ann. N. Y. Acad. Sci.* **102**, 536–548 (1963).
6. van Uden, N. Transport-limited growth in the chemostat and its competitive inhibition; A theoretical treatment. *Arch. Mikrobiol.* **58**, 145–154 (1967).
7. Wallace, R. J. & Holms, W. H. Maintenance coefficients and rates of turnover of cell material in Escherichia coli ML308 at different growth temperatures. *FEMS Microbiol. Lett.* **37**, 317–320 (1986).
8. Nanchen, A., Schicker, A. & Sauer, U. Nonlinear dependency of intracellular fluxes on growth rate in miniaturized continuous cultures of Escherichia coli. *Appl. Environ. Microbiol.* **72**, 1164–1172 (2006).
9. Kovárová, K., Zehnder, A. J. & Egli, T. Temperature-dependent growth kinetics of Escherichia coli ML 30 in glucose-limited continuous culture. *J. Bacteriol.* **178**, 4530 LP – 4539 (1996).
10. Kovárová-Kovar, K. & Egli, T. Growth kinetics of suspended microbial cells: from single-substrate-controlled growth to mixed-substrate kinetics. *Microbiol. Mol. Biol. Rev.* **62**, 646–666 (1998).
11. Schmidt, S. K., Alexander, M. & Shuler, M. L. Predicting threshold concentrations of organic substrates for bacterial growth. *J. Theor. Biol.* **114**, 1–8 (1985).
12. Rittmann, B. E. & McCarty, P. L. Evaluation of steady-state-biofilm kinetics. *Biotechnol. Bioeng.* **22**, 2359–2373 (1980).
13. Senn, H., Lendenmann, U., Snozzi, M., Hamer, G. & Egli, T. The growth of Escherichia coli in glucose-limited chemostat cultures: a re-examination of the kinetics. *Biochim. Biophys. Acta (BBA)-General Subj.* **1201**, 424–436 (1994).
14. Basan, M. *et al.* Overflow metabolism in Escherichia coli results from efficient proteome allocation. *Nature* **528**, 99–104 (2015).
15. Rohwer, J. M., Meadow, N. D., Roseman, S., Westerhoff, H. V & Postma, P. W. Understanding Glucose Transport by the Bacterial Phosphoenolpyruvate:Glycose Phosphotransferase System on the Basis of Kinetic Measurements in Vitro. *J. Biol. Chem.* **275**, 34909–34921 (2000).
16. Gosset, G. Improvement of Escherichia coli production strains by modification of the phosphoenolpyruvate:sugar phosphotransferase system. *Microb. Cell Fact.* **4**, 14 (2005).
17. Jahreis, K., Pimentel-Schmitt, E. F., Brückner, R. & Titgemeyer, F. Ins and outs of glucose transport systems in eubacteria. *FEMS Microbiol. Rev.* **32**, 891–907 (2008).
18. Ferenci, T. Adaptation to life at micromolar nutrient levels: the regulation of Escherichia coli glucose transport by endoinduction and cAMP. *FEMS Microbiol. Rev.* **18**, 301–317 (1996).
19. Hanly, T. J. & Henson, M. A. Dynamic flux balance modeling of microbial co-cultures for efficient batch fermentation of glucose and xylose mixtures. *Biotechnol. Bioeng.* **108**, 376–385 (2011).
20. Postma, P. W. & Roseman, S. The bacterial phosphoenolpyruvate: sugar phosphotransferase system. *Biochim. Biophys. Acta (BBA)-Reviews Biomembr.* **457**, 213–257 (1976).

21. Wong, P., Gladney, S. & Keasling, J. D. Mathematical model of the lac operon: inducer exclusion, catabolite repression, and diauxic growth on glucose and lactose. *Biotechnol. Prog.* **13**, 132–143 (1997).

22. Stock, J. B., Waygood, E. B., Meadow, N. D., Postma, P. W. & Roseman, S. Sugar transport by the bacterial phosphotransferase system. The glucose receptors of the Salmonella typhimurium phosphotransferase system. *J. Biol. Chem.* **257**, 14543–14552 (1982).

23. Bren, A., Hart, Y., Dekel, E., Koster, D. & Alon, U. The last generation of bacterial growth in limiting nutrient. *BMC Syst. Biol.* **7**, 27 (2013).

24. Koch, A. L. & Houston Wang, C. How close to the theoretical diffusion limit do bacterial uptake systems function? *Arch. Microbiol.* **131**, 36–42 (1982).

25. Zeng, H. & Yang, A. Modelling overflow metabolism in Escherichia coli with flux balance analysis incorporating differential proteomic efficiencies of energy pathways. *BMC Syst. Biol.* **13**, 3 (2019).

26. Liu, Y. Overview of some theoretical approaches for derivation of the Monod equation. *Appl. Microbiol. Biotechnol.* **73**, 1241–1250 (2007).

27. Holms, H. Flux analysis and control of the central metabolic pathways in Escherichia coli. *FEMS Microbiol. Rev.* **19**, 85–116 (1996).
